# Performance of Clinical Screening Algorithms for Tuberculosis Intensified Case Finding among People Living with HIV in Western Kenya

**DOI:** 10.1371/journal.pone.0167685

**Published:** 2016-12-09

**Authors:** Surbhi Modi, Joseph S. Cavanaugh, Ray W. Shiraishi, Heather L. Alexander, Kimberly D. McCarthy, Barbara Burmen, Hellen Muttai, Chad M. Heilig, Allyn K. Nakashima, Kevin P. Cain

**Affiliations:** 1 Division of Global HIV & TB, United States Centers for Disease Control and Prevention (CDC), Atlanta, Georgia, United States of America; 2 Division of Tuberculosis Elimination, CDC, Atlanta, Georgia, United States of America; 3 Kenya Medical Research Institute (KEMRI) Center for Global Health Research, Kisumu, Kenya; 4 KEMRI/CDC Research and Public Health Collaboration, Kisumu, Kenya; 5 CDC, Kisumu, Kenya; University College London, UNITED KINGDOM

## Abstract

**Objective:**

To assess the performance of symptom-based screening for tuberculosis (TB), alone and with chest radiography among people living with HIV (PLHIV), including pregnant women, in Western Kenya.

**Design:**

Prospective cohort study

**Methods:**

PLHIV from 15 randomly-selected HIV clinics were screened with three clinical algorithms [World Health Organization (WHO), Ministry of Health (MOH), and “Improving Diagnosis of TB in HIV-infected persons” (ID-TB/HIV) study], underwent chest radiography (unless pregnant), and provided two or more sputum specimens for smear microscopy, liquid culture, and Xpert MTB/RIF. Performance of clinical screening was compared to laboratory results, controlling for the complex design of the survey.

**Results:**

Overall, 738 (85.6%) of 862 PLHIV enrolled were included in the analysis. Estimated TB prevalence was 11.2% (95% CI, 9.9–12.7). Sensitivity of the three screening algorithms was similar [WHO, 74.1% (95% CI, 64.1–82.2); MOH, 77.5% (95% CI, 68.6–84.5); and ID-TB/HIV, 72.5% (95% CI, 60.9–81.7)]. Sensitivity of the WHO algorithm was significantly lower among HIV-infected pregnant women [28.2% (95% CI, 14.9–46.7)] compared to non-pregnant women [78.3% (95% CI, 67.3–86.4)] and men [77.2% (95% CI, 68.3–84.2)]. Chest radiography increased WHO algorithm sensitivity and negative predictive value to 90.9% (95% CI, 86.4–93.9) and 96.1% (95% CI, 94.4–97.3), respectively, among asymptomatic men and non-pregnant women.

**Conclusions:**

Clinical screening missed approximately 25% of laboratory-confirmed TB cases among all PLHIV and more than 70% among HIV-infected pregnant women. National HIV programs should evaluate the feasibility of laboratory-based screening for TB, such as a single Xpert MTB/RIF test for all PLHIV, especially pregnant women, at enrollment in HIV services.

## Introduction

Tuberculosis (TB) remains the leading preventable cause of morbidity and mortality among people living with HIV (PLHIV) [1]. In 2015, 1.2 million (11%) of 10.4 million people who developed TB were HIV-infected, and 390,000 deaths among PLHIV with TB accounted for more than one-fifth of all TB-associated deaths. More than 35% of all HIV-related TB deaths in 2015 occurred in women [2]. If not adequately controlled, TB has the potential to undermine the great strides made globally in rapidly expanding life-saving HIV care and treatment. TB intensified case finding (ICF) is a critical component of the World Health Organization (WHO) recommendations for TB/HIV collaborative activities [3].

In 2010, WHO conducted meta-analysis of existing data on TB screening among PLHIV in 2010 in order to identify an evidence-based clinical screening algorithm. This meta-analysis identified the presence of current cough of any duration, fever, night sweats, or weight loss as the best performing screening rule, with an overall sensitivity of 78.9% for TB among all PLHIV and 90.1% among those screened in clinical settings and a negative predictive value of 95.3% among PLHIV with a 10% prevalence of TB [4]. Based on this evidence, WHO recommends use of this algorithm for screening PLHIV at every clinical encounter [5]. At the time of study implementation, limited data were available about the performance of the WHO algorithm in sub-Saharan Africa. Although a few prospective studies have since evaluated the performance of the WHO clinical screening algorithm for TB among PLHIV, the majority of studies have not assessed implementation of screening by healthcare workers routinely providing care to PLHIV and even fewer have assessed the performance of screening among pregnant women [6–11]. In this paper, we describe our evaluation of the performance of routine TB ICF algorithms among PLHIV newly enrolling in HIV services, including prevention of mother-to-child HIV transmission (PMTCT) services, in a high HIV and TB burden region of Western Kenya.

## Methods

### Study Design and Participants

We conducted a prospective cohort study in Western Kenya to evaluate the performance of clinical screening for TB among adults and older children living with HIV using the WHO TB ICF algorithm [5]. Additionally, we evaluated the performance of the 2009 Kenya Ministry of Health (MOH) ICF algorithm, which was the standard of care for clinical screening in Kenya at the time of this study, and we evaluated the performance of the screening algorithm derived from the “Improving Diagnosis of TB in HIV-infected persons” (ID-TB/HIV) study of PLHIV in three countries in Southeast Asia [12, 13]. The ID-TBHIV study algorithm was one of the first evidence-based clinical screening algorithms for TB among PLHIV, but the performance of the algorithm in sub-Saharan Africa was unknown.

Detailed study procedures have been described elsewhere [14]. Briefly, the sample frame included all public HIV care and treatment facilities (including associated PMTCT services) with at least 200 enrolled patients in the Siaya, Bondo, and Kisumu East Districts of the Nyanza Province. Sites were divided into two strata: small (200–1000 patients; N = 14) and large (>1000 patients; N = 10). Participants were recruited from 15 randomly selected HIV clinics (6 large and 9 small). The number of sites selected from each stratum was proportional to the size of the stratum. Our target sample size was 1000 participants, which accounted for loss to follow-up and was calculated using the Clopper-Pearson method based on assumptions of an expected false-negative screening frequency of 3% based on ID-TB/HIV study findings [12]. Enrollment occurred in a phased manner between May 2011 and June 2012, with each clinical site enrolling participants for 10 weeks. Inclusion criteria were documented HIV infection based on Kenya national guidelines [15], age 7 years or older, and willingness to participate in the study. Exclusion criteria were receipt of any HIV-related care in the preceding two years and TB treatment at enrollment or at any time in the previous one year. Children younger than age 7 years were excluded because of challenges with spontaneous sputum expectoration and the need for alternative diagnostic investigations in this population.

### Clinical Screening and Evaluation for TB

All PLHIV received standard medical care per Kenya MOH guidelines, which included TB screening at entry into care using the 2009 Kenya MOH ICF algorithm [cough ≥ 2 weeks, history of close contact with person with confirmed TB or chronic cough, fever ≥ 2 weeks, noticeable weight loss, chest pain or breathlessness, night sweats ≥2 weeks, swelling in neck, armpit, abdomen, joints or groin] a physical examination, and CD4 count analysis to determine antiretroviral treatment (ART) eligibility [13, 16, 17]. Additionally, all PLHIV were screened for TB at enrollment using the WHO screening algorithm [current cough, fever in the previous 4 weeks, night sweats in the previous 4 weeks, or weight loss in the previous 4 weeks] and an algorithm derived from the “Improving Diagnosis of TB in HIV-infected persons” (ID-TB/HIV) study of PLHIV in three countries in Southeast Asia [any cough in previous 4 weeks, any fever in the previous 4 weeks, or night sweats lasting longer than 3 weeks] [5, 17]. After the clinical screening and regardless of symptoms, PLHIV were referred for chest radiography and asked to provide three sputum specimens within 14 days, including one morning and two spot specimens; specimens were collected over the course of two days. In accordance with local clinical practice, pregnant women were excluded from receiving chest radiography. All medical care, including TB screening, interpretation of chest radiographs, and treatment decisions, was provided in accordance with standard clinical practice by a combination of physicians and non-physicians who were routinely working at the HIV care and treatment facilities. Our study involved assessment for TB disease only; alternative diagnoses were investigated as part of routine medical services and were not captured as part of this study.

### Laboratory Procedures

Sputum specimens were collected at study sites and transported to the Kenya Medical Research Institute (KEMRI)/U.S. Centers for Disease Control and Prevention (CDC) reference laboratory for smear microscopy, mycobacterial culture, and Xpert MTB/RIF (Cepheid Inc., Sunnyvale, CA, USA) [18]. Laboratory personnel were not aware of the clinical signs or symptoms of the individuals who produce the sputum. Xpert MTB/RIF was performed on a 1 ml aliquot of the morning sputum specimen and on the entire second spot specimen (up to 4 mls per manufacturer recommendations). The first spot sputum specimen and the remainder of the morning sputum specimen were cultured using the BACTEC Mycobacteria Growth Indicator Tube (MGIT) 960 system (Becton Dickinson, Sparks, MD, USA) using methods previously described [19]. Positive cultures were identified as *Mycobacterium tuberculosis* complex (MTBC) by Ziehl-Neelson acid fast bacilli (AFB) microscopy and either the Capilia TB Neo (Tauns Laboratories, Inc., Shizuoka, Japan) or the MGIT TBc ID (Becton Dickinson, Sparks, MD, USA) immunochromatographic assay. The Hain Genotype CM line probe assay (Hain Lifescience, Nehren, Germany) was used to further identify culture isolates with non-tuberculous mycobacteria.

### Definitions

PLHIV who reported any symptom or sign in the algorithm suggestive of TB were defined as having “presumptive TB” (previously known as a “TB suspect”) by that algorithm [20]. PLHIV who did not submit at least two sputum specimens or who did not have at least two valid results were excluded. Invalid test results were defined as a contaminated culture or an Xpert MTB/RIF result of error, invalid, or no result. Among the remaining PLHIV, a pulmonary TB case was defined as any person with MTBC confirmed by at least one Xpert MTB/RIF or liquid culture test. PLHIV for whom no sputum specimens were positive for MTBC by Xpert MTB/RIF or liquid culture were considered not to have TB.

### Data Collection and Analysis

Demographic information, clinical symptom screening, and physical examination findings were documented in paper-based medical records by clinicians at each site. Study personnel entered these data into an SQL database, which was merged with the KEMRI/CDC laboratory SQL database. Data were analyzed using SAS version 9.3 (SAS Institute Inc., Cary, NC, USA) and Stata 13.1 (StataCorp. 2013. Stata Statistical Software: Release 13. College Station, TX: StataCorp LP). Symptom screening results were reviewed and recoded for internal consistency so that, for example, patients reporting cough lasting for 2 weeks or longer were also reported as having any cough. We calculated the sensitivity, specificity, negative predictive value, and positive and negative likelihood ratios of the three TB screening algorithms [WHO, MOH and ID-TB/HIV] compared to laboratory-confirmed pulmonary TB. Analyses were weighted and controlled for the complex design of the survey (i.e., clustering, stratification, weighting). Analyses incorporated the use of a finite population correction (FPC) factor to account for the large sampling fraction. The chi-squared tests incorporated a Rao-Schott second order correction to account for the survey design. Differences in age (natural log transformed) and CD4 (square root transformed) were assessed using survey adjusted t-tests.

### Funding and Ethical Review

Funding for this study was provided by the U.S. President’s Emergency Plan for AIDS Relief through Cooperative Agreement 5U19GH000041 from CDC and by the United States Agency for International Development. Ethical approval was obtained from the KEMRI Ethical Review Committee and the CDC Institutional Review Board. We received a waiver of formal written informed consent for participation in this study because (1) the data and specimen collection were not experimental (i.e. they were already recommended as part of Kenyan national guidelines for care of PLHIV); (2) the study activities posed no more than minimal risk to study participants; (3) participation did not adversely affect the welfare or rights of the patients in any way; and (4) to require formal written consent would have imposed an undue burden on the clinical staff of these busy clinics.

## Results

**Fig 1:**
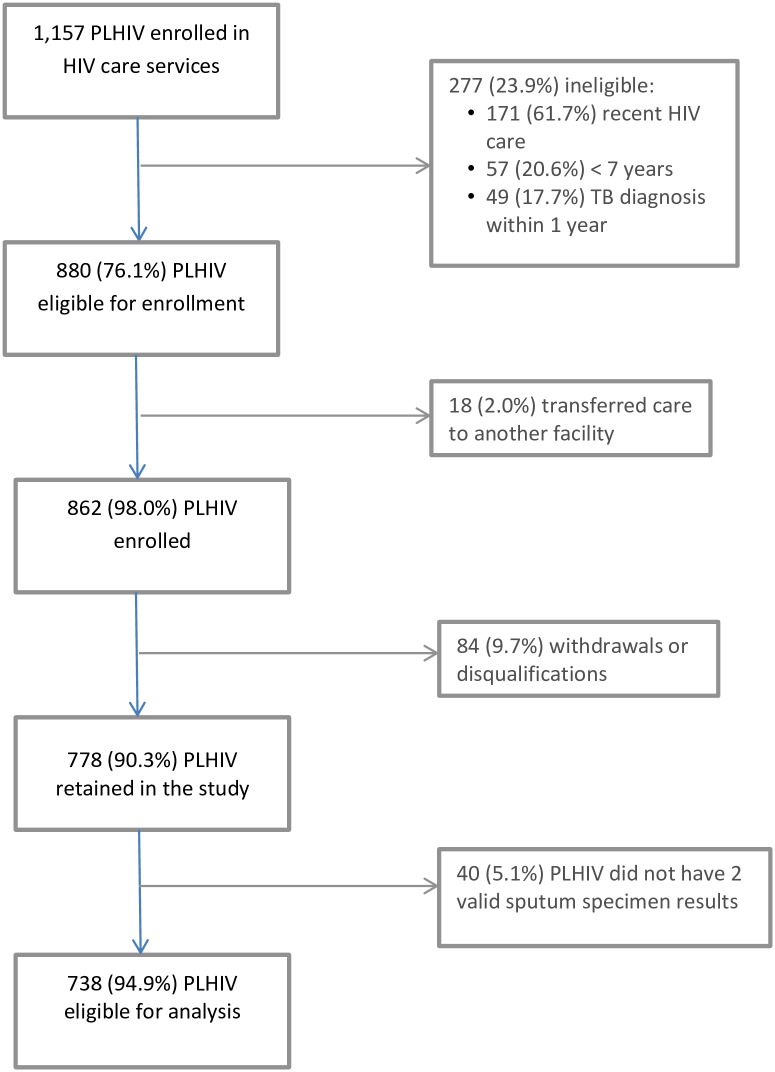
Flow Diagram Showing Numbers of People Living with HIV (PLHIV) Screened, Eligible, Enrolled, and Included in the Analysis.

Between May 2011 and June 2012, 1,157 PLHIV were enrolled in HIV care and treatment at the 15 study sites. Of these, 880 (76.1%) were eligible for enrollment, of which 862 (98.0%) were enrolled (Fig 1). After enrollment, 84 (9.7%) PLHIV were determined to be ineligible or withdrew. An additional 40 (5.1%) PLHIV were excluded because they did not have two valid test results for their sputum specimens, leaving 738 PLHIV for the analysis. No adverse events were reported as part of this study.

Among PLHIV evaluated for TB at enrollment into HIV services, 83 [11.2%; 95% confidence interval (95% CI), 9.9–12.7]) were diagnosed with bacteriologically-confirmed pulmonary TB. The median age of enrolled PLHIV was 30 years (interquartile range, 24–39); 20 (2.7%; 95% CI, 2.1–3.4) PLHIV were younger than 15 years and 487 PLHIV (66.1%; 95% CI, 61.7–70.2) were female. The median CD4+ cell count was 343 per μL (interquartile range, 168–518). Compared to PLHIV in whom no TB diagnosis was made, PLHIV with TB disease had a significantly lower median CD4+ cell count and were significantly more likely to have an abnormal chest radiograph at enrollment (Table 1).

**Table 1:** Characteristics of People Living with HIV (PLHIV) According to Tuberculosis (TB) Diagnosis.

Characteristic	All PLHIV (N = 738), % [95% CI]	TB Prevalence, % [95% CI]	TB Diagnosed (n = 83, 11.2% [9.9, 12.7]), Column % [95% CI]	TB Not Diagnosed (n = 656, 88.8% [87.3, 90.1]), Column % [95% CI]	P value
**Median age (IQR)**	30 (24–39)	----	32 (25–40)	29 (24–39)	0.668
<15 (n = 20)	2.7 [2.1,3.4]	20.1 [11.1, 33.5]	4.8 [2.4, 9.2]	2.4 [1.8, 3.2]	
15–24 (n = 170)	23.1 [20.7, 25.8]	7.1 [5.2, 9.5]	14.5 [11.5, 18.2]	24.2 [21.5, 27.1]	
25–29 (n = 177)	24 [21.8, 26.4]	10.7 [8.1, 14.0]	22.9 [18.1, 28.6]	24.2 [21.9, 26.6]	
30–39 (n = 195)	26.5 [24.1, 29.0]	13.8 [11.7, 16.3]	32.6 [26.8, 38.8]	25.7 [23.6, 28.0]	
40–49 (n = 91)	12.3 [10.2, 14.8]	15.4 [11.1, 20.9]	16.8 [11.1, 24.6]	11.7 [9.6, 14.2]	
50+ (n = 85)	11.4 [9.5, 13.7]	8.3 [4.5, 14.8]	8.4 [4.8, 14.5]	11.8 [9.7, 14.3]	
**Sex**					
Male (n = 251)	33.9 [29.8, 38.3]	13.6 [11.4, 16.1]	40.9 [33.8, 48.4]	33 [28.9, 37.3]	0.007
Female (n = 487)	66.1 [61.7, 70.2]	10.1 [8.6, 11.8]	59.1 [51.6, 66.2]	67 [62.7, 71.1]	
**Pregnant Women**†					
Yes (n = 134)	29.2 [21.8, 37.8]	5.9 [3.6, 9.7]	17.5 [9.7, 29.7]	30.4 [22.8, 39.3]	0.004
No (n = 332)	70.8 [62.2, 78.2]	11.5 [9.6, 13.7]	82.5 [70.3, 90.3]	69.6 [60.7, 77.2]	
**Median CD4 count (IQR)**‡	343 [168, 518]	----	164 [73, 311]	360 [197, 536]	<0.001
<100 (n = 111)	16.1 [14.0, 18.5]	25.1 [19.7, 31.4]	35.7 [29.9, 42.0]	13.6 [11.3, 16.3]	
100–199 (n = 87)	12.7 [11.1, 14.4]	18.5 [14.4, 23.5]	20.7 [14.5, 28.6]	11.6 [10.2, 13.3]	
200–349 (n = 152)	22 [20.6, 23.5]	9.9 [7.0, 13.8]	19.3 [14.5, 25.2]	22.4 [20.9, 23.9]	
350–499 (n = 153)	22.1 [20.4, 24.0]	6.6 [4.0, 10.5]	12.8 [81, 19.6]	23.3 [21.5, 25.3]	
> = 500 (n = 187)	27.1 [25.3, 28.9]	4.8 [3.2, 7.1]	11.4 [8.0, 16.1]	29.1 [26.9, 31.3]	
**Symptoms in World Health Organization algorithm**§					
Current cough (n = 173)	24.7 [21.1, 28.8]	18.6 [15.7, 21.8]	42.7 [33.4, 52.7]	22.6 [19.3, 26.2]	<0.001
No current cough (n = 526)	75.3 [71.2, 78.9]	8.2 [6.6, 10.1]	57.3 [47.3, 66.6]	77.4 [73.8, 80.7]	
Fever in previous 4 weeks (n = 240)	33.6 [29.6, 37.9]	17.9 [15.6, 20.5]	54.3 [44.3, 64.0]	31 [27.4, 34.8]	<0.001
No fever in previous 4 weeks (n = 472)	66.4 [62.1, 70.4]	7.6 [5.9, 9.8]	45.7 [36.0, 55.7]	69 [65.2, 72.6]	
Night sweats in previous 4 weeks (n = 185)	26.2 [22.0, 30.8]	19.5 [16.1, 23.4]	46.3 [34.8,58.2]	23.7 [19.9,27.9]	<0.001
No night sweats in previous 4 weeks (n = 522)	73.8 [69.2, 78.0]	8 [6.2, 10.3]	53.7 [41.8,65.2]	76.3 [72.1,80.1]	
Weight loss in previous 4 weeks (n = 244)	34 [28.8,39.6]	18.5 [15.2,22.3]	57.6 [48.8,66.0]	31.1 [25.9,36.8]	<0.001
No weight loss in previous 4 weeks (n = 471)	66 [60.4,71.2]	7 [5.7,8.6]	42.4 [34.0,51.2]	68.9 [63.2,74.1]	
**Any lymphadenopathy**€					
Yes (n = 36)	5.1 [3.7, 7.0]	16.9 [10.8, 25.4]	7.6 [5.1, 11.2]	4.7 [3.2, 6.9]	0.074
No (n = 669)	94.9 [93.0, 96.3]	10.9 [9.3, 12.7]	92.4 [88.8, 94.9]	95.3 [93.1, 96.8]	
Chest radiograph**					
Normal (n = 319)	55.8 [47.5, 63.7]	5.4 [4.0, 7.2]	24.3 [17.8, 32.2]	60.2 [51.1, 68.6]	<0.001
Abnormal (n = 253)	44.2 [36.3, 52.5]	21 [17.1, 25.5]	75.7 [67.8, 82.2]	39.8 [31.2, 48.9]	
**Abnormal chest radiograph**§§					
Consistent with TB (n = 102)	41.3 [34.9, 47.9]	39.2 [31.0, 48.1]	75.5 [65.2, 83.6]	31.9 [24.9, 39.9]	<0.001
Not Consistent with TB (n = 146)	58.7 [52.1, 65.1]	8.9 [5.7, 13.6]	24.5 [16.4, 34.8]	68.1 [60.1, 75.1]	

*The n reported for each characteristic refers to PLHIV and is not provided for each column

^†^ Excludes 21 women whose pregnancy status was unknown

^‡^Excludes 48 people with missing CD4 count at enrollment, 5 of whom were diagnosed with TB and 44 of whom did not have TB

^§^ Excludes 39 PLHIV without assessment of current cough, 26 PLHIV without assessment of fever in the previous 4 weeks, 31 PLHIV without assessment of night sweats in the previous 4 weeks, and 23 PLHIV without assessment of weight loss in the previous 4 weeks

^€^ Excludes 33 PLHIV in whom lymphadenopathy was not assessed

** Excludes 166PLHIV who did not have a chest radiograph, including 134 pregnant women who were not referred for chest radiography

^§§^ Excludes 5 PLHIV with abnormal chest radiographs which were not classified as consistent or not consistent with TB

### Performance of the WHO Screening Algorithm among All PLHIV Enrolling in HIV Care

Documentation of TB screening using the WHO screening algorithm was complete for 696 [94.3% (unweighted proportion)] of 738 PLHIV. Overall, 53.2% of PLHIV screened at enrollment reported having at least one symptom in the WHO screening algorithm (Table 2). Among PLHIV with TB disease, 74.1% [95% CI, 64.1–82.2] had a positive WHO symptom screen (90.3% [95% CI, 79.1–95.8] for smear-positive TB disease and 63.0% [95% CI, 54.3–70.9] for smear-negative disease) compared to 50.5% [95% CI, 46.1–54.9] PLHIV who did not have TB. Weight loss in the previous 4 weeks and fever were commonly reported symptoms among all PLHIV [34% (95%CI, 28.8–39.6) and 33.6% (95% CI, 29.6–37.9), respectively] and among PLHIV with TB [57.6% (95% CI, 48.8–66.0) and 54.3% (95% CI, 44.3–64.0), respectively]. Current cough was reported by 42.7% (95% CI, 33.4–52.7) of PLHIV with TB disease compared to 24.7% (95% CI, 21.1–28.8) of PLHIV without TB disease (Table 1).

**Table 2:** Performance of World Health Organization (WHO) Tuberculosis (TB) Intensified Case Finding Algorithm in People Living with HIV (PLHIV) in Kenya.

	Percent Screening Positive [95% CI]	Sensitivity [95% CI]	Specificity [95% CI]	Negative Predictive Value [95% CI]	Positive Predictive Value [95% CI]	Likelihood Ratio Positive [95% CI]	Likelihood Ratio Negative [95% CI]
WHO Algorithm*	53.2 [48.5,57.8]	74.1 [64.1,82.2]	49.5 [45.1,53.9]	93.8 [91.4,95.6]	15.6 [13.8,17.7]	1.47 [1.38, 1.57]	0.53 [0.40, 0.68]
**WHO Algorithm + Chest Radiograph**†	70.8 [66.5,74.8]	90.9 [86.4,93.9]	32 [27.5,36.8]	96.1 [94.4,97.3]	15.8 [13.3,18.6]	1.34 [1.25, 1.42]	0.29 [0.20, 0.41]
**WHO Algorithm + Contact with TB case**‡	53.6 [47.5,59.6]	77.5 [68.6,84.5]	49.4 [43.3,55.5]	94.7 [92.9,96.0]	15.9 [13.6,18.6]	1.53 [1.44, 1.63]	0.46 [0.36, 0.58]
**WHO Algorithm Performance for Age< 15 years**§	84.7 [72.3,92.2]	100 --	19.4 [9.6,35.3]	100 --	24.9 [13.5,41.4]	1.24 [1.07, 1.43]	--
**WHO Algorithm Performance for Adults (≥ 15 years)**§	52.3 [47.7,56.9]	72.8 [62.8,80.9]	50.2 [45.8,54.6]	93.8 [91.3,95.5]	15.2 [13.2,17.5]	1.46 [1.36, 1.57]	0.54 [0.43, 0.69]
**WHO Algorithm Performance for PLHIV with CD4 < 350**€	67.7 [63.5,71.6]	77.6 [68.7,84.5]	34.3 [30.5,38.3]	88.5 [84.2,91.8]	19 [16.0,22.4]	1.18 [1.09, 1.28]	0.65 [0.49, 0.87]
**WHO Algorithm Performance for PLHIV with CD4 ≥350** €	39.7 [34.5,45.2]	57.6 [41.8,72.0]	61.4 [56.2,66.3]	95.9 [93.3,97.5]	8.5 [6.4,11.2]	1.49 [1.26, 1.72]	0.69 [0.52, 0.91]
**WHO Algorithm Performance Stratified by Sex and Pregnancy¥**							
Pregnant Women	25.6 [19.7,32.5]	28.2 [14.9,46.7]	74.6 [67.6,80.4]	94.8 [90.4,97.3]	5.9 [2.8,11.9]	1.11 [0.68, 1.80]	0.96 [0.80, 1.17]
Non-Pregnant Women	58.1 [54.4,61.8]	78.3 [67.3,86.4]	44.5 [40.9,48.3]	93.9 [90.3,96.2]	15.8 [13.0,19.0]	1.41 [1.26, 1.58]	0.49 [0.33, 0.72]
Men	61.8 [56.7,66.6]	77.2 [68.3, 84.2]	40.6 [35.4, 46.1]	92.0 [88.1, 94.6]	16.8 [14.1,20.0]	1.30 [1.16, 1.46]	0.56 [0.41, 0.78]

*Excludes 42 people with incomplete TB symptom screening data

^†^ Excludes 42 people with incomplete TB symptom screening data and an additional 157 with no chest radiograph (including all 134 pregnant women)

^‡^ Excludes 42 people with incomplete TB symptom screening data and an additional 7 with no data on TB contact

^§^ Excludes 1 child younger than 15 years and 42 adults with incomplete TB symptom screening data and an additional 47 adults with missing CD4 count at enrollment

^€^ Excludes 42 people with incomplete TB symptom screening data and an additional 19 with missing pregnancy status

Among 696 PLHIV with documentation of WHO screening, 539 [77.4% (unweighted proportion)] received chest radiography. Adding a chest radiograph to the screening algorithm so that PLHIV with any symptom or an abnormal chest radiograph were classified as screening positive increased sensitivity compared to WHO symptom screening alone [90.9% (95% CI, 86.4–93.9) versus 74.1% (95% CI, 64.1–82.2)], slightly increased negative predictive value [96.1% (95% CI, 94.4–97.3) versus 93.8% (95% CI, 91.4–95.6)], but decreased specificity [32.0% (95% CI, 27.5–36.8) versus 49.5% (95% CI, 45.1–53.9)] (Table 2). Among PLHIV with a CD4+ cell count below 100 cells/μL at enrollment, the WHO screening algorithm had a sensitivity of 92.6% (95% CI, 83.8–96.8), although specificity decreased to 24.3% (95% CI, 20.3–28.9) and negative predictive value decreased to 90.6% (95% CI, 78.9–96.2) (data not shown).

### Performance of Kenya MOH and ID-TB/HIV Clinical Screening Algorithms among All PLHIV Enrolling in HIV Care

Overall, 50.1% of all PLHIV reported having at least one of the symptoms in the MOH TB screening algorithm, with weight loss remaining the most commonly reported symptom (66.0% of PLHIV). This algorithm performed as follows: sensitivity 77.5% (95% CI, 68.6–84.5), specificity 49.4% (95% CI, 43.3–55.5), and negative predictive value of 94.7% (95% CI, 92.9–96.0). The ID-TB/HIV algorithm performed similarly, with a sensitivity of 72.5% (95% CI, 60.9–81.7), specificity 56.5% (95% CI, 52.5–60.5), and negative predictive value of 94.3% (95% CI, 91.6–96.2) [data not shown].

### Screening for TB among Pregnant Women Enrolling in PMTCT Services

Among the 487 women enrolled in this study, 134 (27.5%) were pregnant women identified at enrollment into PMTCT services. These pregnant women represented 18.2% of all PLHIV in this study and had a median age of 25 years (interquartile range, 22–29). TB prevalence among pregnant women was lower than among both non-pregnant women and men [5.9% (95% CI, 3.6–9.7) versus 11.5% (95% CI, 9.6–13.7) and 13.6% (95% CI, 11.4–16.1), respectively]. Pregnant women with TB did not have a significantly different baseline median CD4+ cell count (377cells/μL; IQR, 165–609cells/μL) than pregnant women without TB (401 cells/μL; IQR, 255, 637 cells/μL). Only 33 (24.6%) pregnant women reported having at least one of the symptoms in the WHO screening algorithm compared to 195 (55.2%) non-pregnant women and 143 (57.0%) men; night sweats was most commonly reported [57.6% (95% CI, 47.4–67.0) of symptomatic pregnant women] and weight loss was least commonly reported [36.1% (95% CI, 26.1–48.6) of symptomatic pregnant women]. The sensitivity of the WHO algorithm was significantly lower among pregnant women, 28.2% [95% CI, 14.9–46.7] compared to 78.3% [95% CI, 67.3–86.4] for non-pregnant women and 77.2% [95% CI, 68.3–84.2] for men (Table 2).

## Discussion

In this study, the prevalence of bacteriologically-confirmed TB among PLHIV enrolling in HIV services was 11.2%. This is consistent with findings from a multi-country study that found a 12% TB prevalence among PLHIV not on ART in four countries in sub-Saharan Africa [10]; the TB prevalence is lower than the 15% TB prevalence among PLHIV in three countries in Southeast Asia in the ID-TB/HIV study, but this may also be related to the lower initial median CD4+ cell count in that study (242 cells/ μL) [12]. In our study population, the three clinical screening algorithms performed similarly and the WHO clinical screening algorithm performed as expected among all PLHIV, given the TB prevalence [4, 5]. However, the performance of clinical screening was variable across several sub-sets of PLHIV, including those who were severely immunosuppressed and pregnant women accessing PMTCT services, which has important programmatic implications.

The prevalence of TB varied across the districts, and likely reflects the burden of disease in those districts. Given the substantial burden of TB among PLHIV at enrollment in HIV care, TB case finding should be a priority intervention in HIV care and treatment and PMTCT settings. Although implementation of routine ICF is expanding, only 7 million (19%) of the 36.9 million PLHIV worldwide in 2014 were reported to be screened for TB [2, 21]. Our study demonstrates that half of all PLHIV newly enrolling in HIV care services reported at least one TB symptom. However, only 15% of symptomatic PLHIV were diagnosed with pulmonary TB, meaning that the majority of PLHIV identified with presumptive TB were not TB cases. Additionally, if TB diagnostic testing were limited to PLHIV reporting symptoms in the WHO algorithm, 25% of all PLHIV with bacteriologically-confirmed pulmonary TB would have been missed. According to WHO guidelines, these asymptomatic PLHIV with TB disease would be candidates for isoniazid preventive therapy, meaning that they would have erroneously received monotherapy instead of the recommended four-drug TB treatment regimen. Repeat clinical screening is recommended for all PLHIV receiving isoniazid preventive therapy to identify those with TB disease who are missed on an initial screen; however, most studies of intensified case finding, including ours, have reported on the yield at entry into HIV services making evidence about the performance of clinical screening during repeat clinical visits limited.

Our results confirm that the performance of the WHO TB ICF algorithm varies with the level of immunocompromise [22, 23]. Clinical screening was more sensitive for TB case finding among PLHIV with a CD4+ count below 100 cells/μL at enrollment, identifying more than 92% of these PLHIV with TB disease as needing a diagnostic evaluation. Because CD4 count results are not available in all settings or are often received after the patient’s first visit to the HIV care and treatment clinic, the utility of CD4 count for identifying priority populations for clinical TB screening or for prioritizing PLHIV with presumptive TB for diagnostic evaluation is limited.

Approximately two-thirds of PLHIV enrolled in this study were women, which is consistent with findings from a review of ART programs which found that the female-to-male new ART enrollee ratios were 2.10 in countries in East Africa, partially because of access to HIV testing and ART as part of antenatal services for pregnant women [24]. Our study found that clinical TB screening was particularly ineffective for TB case finding among pregnant women accessing PMTCT services. Half as many pregnant women reported TB symptoms at enrollment in HIV care as other PLHIV; if TB diagnostic testing were limited to pregnant women reporting symptoms in the WHO algorithm, more than two-thirds of laboratory-confirmed pulmonary TB would have been missed. These findings are consistent with data from other studies of TB screening among pregnant women living with HIV [11, 25–28]. One possible explanation for this difference is that pregnancy “masks” the symptoms of TB, making common TB symptoms such as weight loss less evident [29]. Indeed, weight loss was the least commonly reported symptom among pregnant women despite being the most commonly reported symptom for all PLHIV. An additional possibility is that pregnant women with TB were screened and tested at an earlier stage of disease than non-pregnant women. Screening tools and diagnostic tests are expected to have lower sensitivity in early- stage disease than in late-stage disease [30]. Although we did not assess reasons for seeking care, PLHIV who were not pregnant may have presented for clinical attention because they were feeling unwell, whereas pregnant women more likely sought clinical care for their pregnancy. If this hypothesis were true, then the difference in sensitivity observed could be due to the different average stage of TB disease present in the two groups. Ultimately, given the unreliability of symptom-based TB screening among HIV-infected pregnant women, alternative strategies, such as Xpert MTB/RIF testing for all pregnant women, are warranted [31]. The high prevalence of TB in our population, combined with the sub-optimal sensitivity of symptom-based screening, would suggest that such a strategy could be considered for the initial evaluation of all patients with HIV.

In this study, TB prevalence among pregnant women living with HIV was approximately half that of non-pregnant women and men living with HIV. National TB surveillance systems do not routinely report pregnancy status of TB cases. However, a recent study estimated that the global burden of TB in pregnancy was substantial, with 216,500 cases in 2011, 41% of which occurred in the WHO AFRO region [32]. Data from the United Kingdom show that TB incidence in the postpartum period is significantly higher than among pregnant women or non-pregnant women outside of the postpartum period, potentially reflecting delays in diagnosis during pregnancy due to diagnostic challenges and immunologic changes [33]. Partially due to the lower prevalence of TB among pregnant women, the negative predictive value of the WHO TB ICF algorithm was comparable among pregnant women and other PLHIV.

Strategies to optimize the performance of TB screening include expanding the number of symptoms and signs included in the screening algorithm, assessing for TB contact status, and adding chest radiograph. In our study, we found that the Kenya MOH TB screening algorithm, which included a combination of six symptoms and close contact with a person with TB disease, only marginally increased sensitivity, suggesting that expanded clinical screening likely has limited value. Including chest radiography as part of the WHO symptom screening algorithm improved case finding and should be considered as feasible, keeping in mind that this increase in sensitivity was associated with a decrease in specificity. This decrease in specificity is not surprising given that PLHIV commonly have non-TB related lung changes which in some cases can complicate radiologic TB diagnosis, especially in early stages of disease [23].

This study had multiple limitations. Symptom screening was conducted as part of routine clinical services and clinical symptoms were not independently verified by study staff. Additionally, under these routine practice conditions, we were unable to obtain complete data on all patients. Clinical information, such as baseline CD4+ cell count and chest radiograph results, was missing for a subset of patients. Approximately 5% of PLHIV enrolled in the study were excluded from the final analysis because they were unable to provide more than one sputum specimen or provided specimens that could not be examined due to culture contamination. Of the 40 excluded PLHIV, 2 (5%) were found to have TB disease by Xpert MTB/RIF or culture of one sputum specimen; exploratory analysis with differing exclusion criteria did not result in substantive differences in the performance of the clinical screening algorithms (data not shown). However, the true prevalence of TB among the other PLHIV with culture contamination is unknown. Finally, while our study was designed to be representative of the Kisumu, Siaya, and Bondo Districts of Kenya as defined at the time of study inception, our study was not designed to be nationally-representative or generalizable to other settings in Kenya or sub-Saharan Africa.

This study confirms that the WHO TB ICF algorithm performs as predicted among PLHIV newly enrolling in HIV services in this high HIV and TB burden region of Kenya. However, as half of all PLHIV reported symptoms consistent with TB disease, use of this clinical screening algorithm would lead to diagnostic evaluation of a large number of PLHIV without TB disease and would additionally miss asymptomatic PLHIV with TB disease. Given the poor performance of clinical screening among pregnant women, national HIV and PMTCT programs should evaluate the programmatic feasibility and cost implications of laboratory-based screening for TB disease at the initial presentation for HIV care, such as requesting a single Xpert MTB/RIF test for all HIV-infected pregnant women and potentially all PLHIV, enrolling in HIV services. Additional analyses are needed to determine the performance of WHO screening at follow-up visits, strategies to improve the sensitivity and negative predictive value of clinical screening algorithms, optimal intervals for screening, and to assess whether different clinical or laboratory-based (e.g. Xpert MTB/RIF) screening algorithms are more sensitive among pregnant women living with HIV.
